# Geographic Transmission and Epidemic History of HIV-1 CRF01_AE, CRF07_BC, and HCV Subtype-6w among Taiwanese Persons Who Inject Drugs

**DOI:** 10.3390/v14102142

**Published:** 2022-09-28

**Authors:** Yen-Ju Chen, Jason C. Huang, Hung-Chin Tsai, Yu-Hui Lin, Kuo-Feng Hsu, Hsin-Fu Liu

**Affiliations:** 1 Research Assistant Center, Tainan Municipal Hospital (Managed by Show Chwan Medical Care Corporation), Tainan 701033, Taiwan; 2 Department of Biotechnology and Laboratory Science in Medicine, National Yang Ming Chiao Tung University, Taipei 112304, Taiwan; 3 Department of Medicine, Kaohsiung Veterans General Hospital, Kaohsiung 813414, Taiwan; 4 Department of Medicine, Taichung Veterans General Hospital, Taichung 40705, Taiwan; 5 Department of Surgery, Tri-Service General Hospital, National Defense Medical Center, Taipei 114202, Taiwan; 6 Department of Medical Research, Mackay Memorial Hospital, Taipei 25169, Taiwan; 7 Institute of Biomedical Sciences, MacKay Medical College, New Taipei City 25245, Taiwan

**Keywords:** geographic transmission, epidemic history, transmission routes, time of the most recent common ancestor (tMRCA), persons who inject drugs (PWID)

## Abstract

Persons who inject drugs (PWID) and their risk-related behaviors (e.g., unprotected sex and sharing needles/syringes/other injection equipment) have caused severe public health problems, especially in the rapid spread of HIV-1 and HCV. Here, we reconstructed the epidemic history of HIV-1 circulating recombinant form (CRF) 01_AE, CRF07_BC, and HCV subtype-6w among Taiwanese PWID. The timescales were estimated using phylogenetic and Bayesian coalescent analyses. The results revealed that CRF01_AE started to circulate in the Taiwanese PWID population in central Taiwan at 1992.5 (95% credible region: 1988.8–1995.9) and spread to other regions of Taiwan, while CRF07_BC was first identified in southern Taiwan at 2000.0 (95% CR: 1997.8–2002.2) and then spread northward to central-northern Taiwan. All HCV-6 strains were from Asia (that is, China, Myanmar, Taiwan, and Vietnam) and originated in 1928.1 (95% CR: 1890.2–1966.0). Furthermore, subtype-6w isolates from different regions of Taiwan appeared to share a common source that existed in the mid-1990s (95% CR: 1985.0–2001.8) or thereabouts. The routes of drug trafficking and the resulting high prevalence of HIV-1/HCV co-infections among PWID might have contributed to the virus transmission and promoted its spread worldwide. Long-term monitoring and policy implementation in at-risk populations would be useful for disease control.

## 1. Introduction

In 2021, 38.4 million people globally were estimated to have lived with HIV and the worst region is still sub-Saharan Africa [1]. A systematic review revealed that of the 16 million people who inject drugs (PWID), one-fourth lived in East and Southeast Asia [2]. PWID and their risk-related behaviors (e.g., unprotected sex and sharing syringes/heroin solutions/other injection equipment) have caused server public health problems, especially regarding the rapid spread of HIV-1 and HCV. According to the distribution data for the HIV-1 subtypes and the circulating recombinant forms (CRFs) in Asia, the HIV-1 subtype B’ and CRF01_AE are the main subtypes among PWID in Thailand [3,4,5], Myanmar [6], and Vietnam [7], while CRF07_BC is the predominant strain among PWID in China and Taiwan [8,9,10,11,12]. The strain CRF07_BC is derived from the Thai subtype B’ and Indian subtype C lineages [13]. Since 1997, CRF07_BC has been isolated from PWID in several provinces of China [8,9]. This strain is presumed to originate in Yunnan province [14,15] and has spread northwest to Xinjiang province and east to Guangxi province [13,16]. In this context, Yunnan plays an important role as an entry point for heroin trafficking into China [17] and is considered an epicenter of HIV/AIDS in China [13]. 

In Taiwan, most HIV-1 infected cases in the PWID were CRF07_BC [10,11,12]. Phylogenetic analyzes revealed that Taiwanese CRF07_BC strains clustered with CRF07_BC isolates from Xinjiang (97CN54, 97CN001, and 98CN009) and Guangxi (CNGL179) provinces of China [10,11,12]. Furthermore, it has been suggested that CRF07_BC initially circulated in southern Taiwan (Tainan) and then to other regions of Taiwan later [10,11]. Although predominate in PWID, CRF07_BC has also been spread to men who have sex with men (MSM) and heterosexuality [11]. Besides CRF07_BC, an outbreak of CRF01_AE infection was identified among PWID in Central-North Taiwan [10,11]. Evidence suggests that CRF01_AE was introduced into Taiwanese PWID through unprotected sex [11]. 

Both HIV-1 and HCV are considered blood-borne diseases, which are transmitted mainly through blood contact with infections. The prevalence of chronic hepatitis C in the Taiwanese population is approximately 2.1%, which was among the countries with the higher prevalence in Asia [18]. Paintsil et al. pointed out that HCV dried on inanimate surfaces can remain infectious for up to six weeks at normal room temperature [19]. Currently, the new HCV infection has shifted to PWID and other high-risk groups, including HIV-infected people and MSM who did not take effective safety measures. Traditional HCV treatment was a combination therapy (interferon injection plus oral ribavirin medicine), which successfully cured 70–80% of genotype 1 and >90% of genotype 2 patients [20,21,22,23,24]. With direct-acting antivirals (DAAs) that were launched in 2014, the successful cure rate increased to 95%. 

During the explosive 2004–2006 outbreak of HIV-1 CRF07_BC among Taiwanese PWID, almost all of them (~99.3%) were HIV-1/HCV co-infections [10,11,25]. Previous studies on the geographical distribution of HCV showed that different dominant types existed throughout the world [26,27] and profound prevalence changes were observed in different genotypes of HCV over time [28,29]. Four predominant subtypes, namely 6a, 1a, 1b, and 3a, were identified in blood specimens from Taiwanese PWID infected with HCV. In particular, subtype-6w (1.4%) was detected at the same time in this subpopulation (see Table A1). Several studies have attempted to reconstruct the epidemic history of HIV-1 outbreaks among PWID in Asia [17,30,31]. In our previous study, we included HIV diagnosis when submitting virus nucleotide sequences to GenBank. To avoid errors in the calculation, we used the specimen collection date for all isolates to estimate the epidemic period. Furthermore, we focused only on PWID infected with HIV-1 CRF01_AE, CRF07_BC, and HCV-6. Since HCV subtype-6w is uncommon and no literature has yet described its transmission routes and studies in Taiwanese IDUs focused only on subtype-6w, we conducted an investigation of HCV to track its transmission routes of subtype-6w and estimate the time of emergence of genotype-6 among Asian PWID. 

## 2. Materials and Methods

### 2.1. Subjects

**Figure A1:**
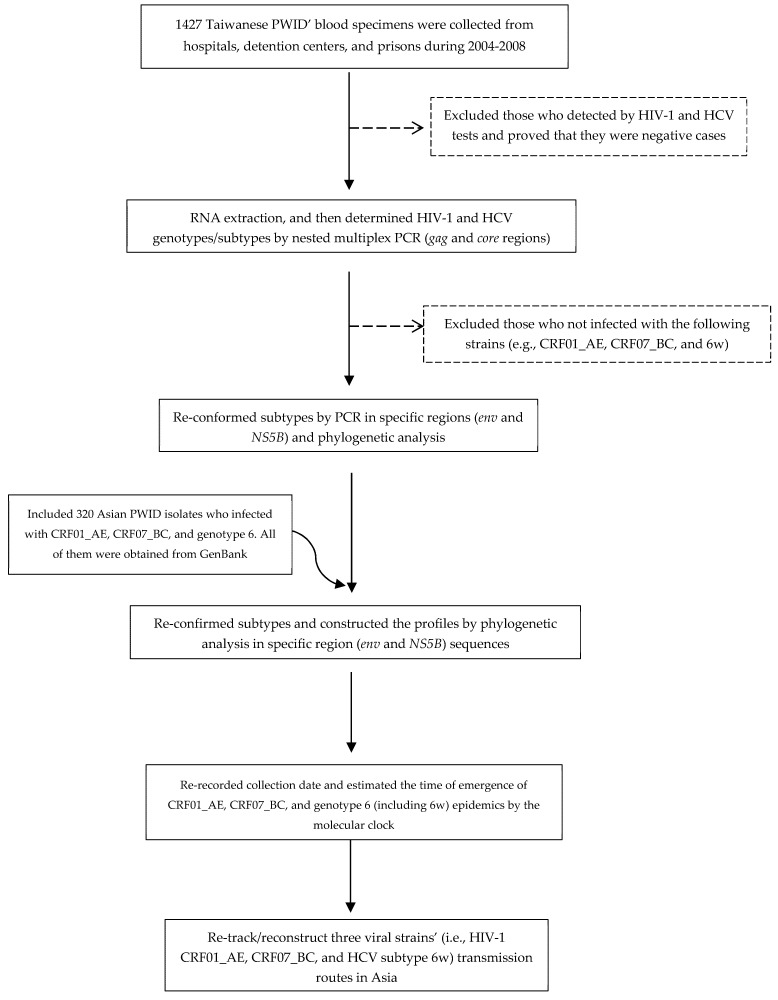
Study flowchart. A total of 1427 Taiwanese PWID blood samples were collected from hospitals, detention centers, and prisons during 2004–2008. Four hundred sequences were used in phylogenetic and evolutionary analyzes.

The research procedures for the current study are shown in Figure A1. A total of 1427 PWID were recruited from Taipei City Hospital, Sindian Drug Abuse Treatment Center, Taipei Detention Center and Prison, Taoyuan Woman’s Prison (Northern Taiwan), Taichung Detention Center and Prison, Yunlin Second Prison, Nantou Detention Center (Central Taiwan), Tainan Detention Center, and Kaohsiung Prison (Southern Taiwan). Among the blood samples collected, 611 cases collected between 2004 and 2005 were shown to be infected with HIV-1 while 9 cases collected between 2005 and 2008 were HCV-6w. To track the routes of Taiwanese HIV-1 CRF01_AE, CRF07_BC, and HCV-6w transmission, we integrated sequences of Asian isolates available from the NCBI database (https://www.ncbi.nlm.nih.gov/nucleotide/, accessed on 8 July 2021) in our evolutionary analysis. All Taiwanese PWID were obtained by direct sequencing in our laboratory and those retrieved from GenBank were listed in Table A2. 

Sociodemographic data and information on the types of illegal drugs used, history of drug abuse, risk factors associated with HIV-1 transmission, and years of the first HIV/HCV positive diagnosis were collected using a self-administered questionnaire. Peripheral blood samples were collected to allow analysis of virus genotype. Informed consent was obtained from all participants. Our research protocol was approved by the prisons and detentions administration system, as well as the Institutional Review Board of the National Yang-Ming University, Taiwan. 

### 2.2. HIV-1 and HCV Subtyping

Viral RNA was extracted from plasma samples using the QIAamp Viral RNA mini kit (QIAGEN, Hilden, Germany). Random primer (Promega) was used in reverse transcription to generate cDNA for reverse transcriptase-polymerase chain reaction (RT-PCR). Anti-HCV antibodies from serum samples were detected using an enzyme immunoassay system (Murex 3nd, Abbott Laboratories, North Chicago, IL, USA). Specimens determined with anti-HCV antibodies or confirmed as HIV-1 positive were further analyzed. The genotypes/subtypes of HCV and HIV-1 infections were determined according to the methods described previously [10,25,32]. A set of primers, OF9-2 (forward) 5′-CGACATTACGCAGAAGTTGCCC-3′ and OR9 (reverse) 5′-AGTGTTGCTTAAGGCCTCCTGC-3′, were used to amplification of the HCV *NS5B* gene near full-length. Proviral nucleotide sequences were obtained by direct sequencing of PCR products using a DNA analyzer (ABI 3730, Applied Biosystems, Foster City, CA, USA). 

### 2.3. Phylogenetic Analysis

**Figure A2:**
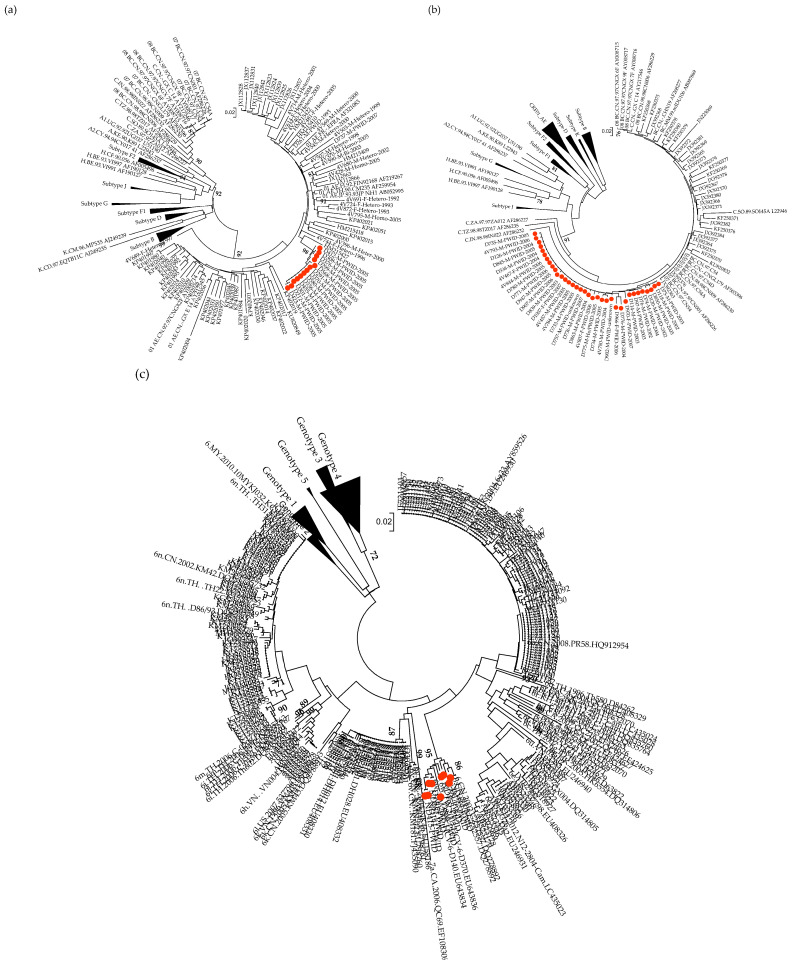
Phylogenetic analysis of HIV-1 CRF01_AE, CRF07_BC, and HCV sequences among Asian PWID. (**a**) CRF01_AE, the NJ tree was constructed using MEGA under the TN93 + G model with 1000 bootstrap replicates. (**b**) CRF07_BC, TN93 + G and (**c**) HCV, K2 + G. The red point indicates the Taiwanese PWID sequences.

Sequence alignment analysis with various reference strains from the Los Alamos HIV-1 database (https://www.hiv.lanl.gov/content/index, accessed on 28 May 2021) and the HCV database (https://hcv.lanl.gov/content/index, accessed on 8 July 2021) was performed using the BioEdit v7.2.6.1 program [33]. The MEGA X program [34] was used to find the best fit nucleotide substitution model and to construct phylogenetic trees using neighbor-joining (NJ) and maximum likelihood (ML) methods. For example, taking the HIV-1 *env* gene, the substitution model GTR + G was incorporated into the ML method, while TN93 + G (GTR and HKY models are not available here) was used to calculate the evolutionary distance for the NJ tree followed by bootstrap analysis with 1000 replicates [35]. Considering the best-fit models for the HCV *NS5B* gene, the substitution models for both the ML and NJ tree were K2 + G + I. At least four strains of all subtypes were used as reference sequences and isolates from Asian PWID were included for phylogenetic analysis (see Figure A2). Bootstrap values (≥70%) were used as an indicator of the significance of the clusters.

### 2.4. Nucleotide Sequence Accession Numbers

The HIV-1 *env* sequences (OM287868–OM287928) and the HCV *NS5B* sequences (OM287929–OM287937) were obtained from the current study and deposited in GenBank. 

### 2.5. Bayesian Coalescent Inference

Evolutionary rates were obtained using the Bayesian Markov chain Monte Carlo (MCMC) approach implemented in BEAST v2.5.1 [36]. General time-reversible (GTR) [37,38,39] substitution models with gamma-distributed among-site rate variation involving six categories [40] were used to estimate evolutionary rates and construct tree topologies. Constantly sized, exponentially growing, and Bayesian skyline coalescent models were used for each case [41] and each MCMC chain was run for at least 10,000,000 states and sampled in every 1000 states. Posterior probability densities were calculated, and the convergence of the chains was verified using the Tracer v1.7.1 [42] with 10% of each chain discarded as burn-in. 

### 2.6. Statistical Analysis

The Pearson χ^2^ test and Fisher’s exact test were performed in univariate analysis of demographic data. The difference between groups with a *p*-value < 0.05 was considered statistically significant. The *p*-values were two-tailed and unadjusted for multiple comparisons. 

## 3. Results

### 3.1. Geographical Distribution of HIV-1 CRF01_AE, CRF07_BC, and HCV Subtype-6w among Taiwanese PWID 

From 2004 to 2005, almost all Taiwanese HIV-1 positive PWID were infected with CRF07_BC. However, we found another small-scale outbreak strain that circulated in this population in central-north Taiwan. These were judged according to their time of crime and the place of sentences.

**Figure A3:**
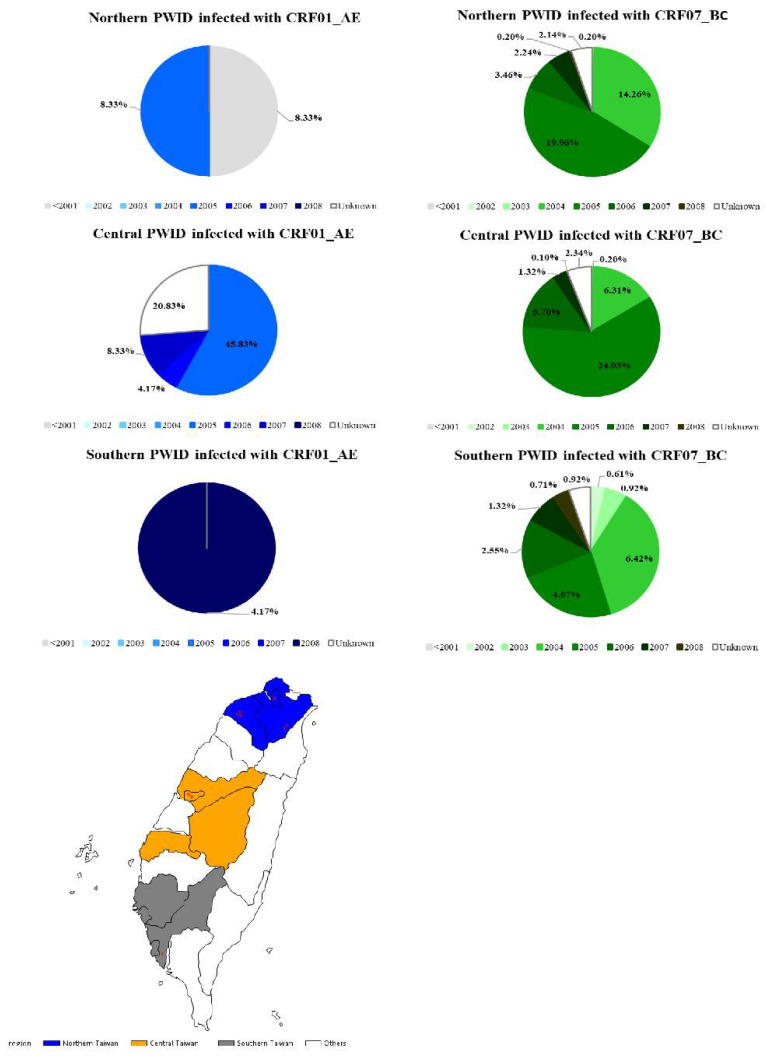
Distribution of Taiwanese PWID infected with HIV-1 CRF01_AE (*n* = 24), HIV-1 CRF07_BC (*n* = 982), and HCV subtype 6w (*n* = 9) during 2004–2008. Each strain was labeled with a different color to denote patient characteristics, including collection place and year of HIV-1 diagnosis. The proportion of viral strains circulated into Northern-Central-Southern Taiwan per year, divided by total cases infected with the same strain, is shown in pie charts. Because Taiwanese PWID infected with HCV-6w were rare, we directly labeled their locations on the map.

As shown in Figure A3, the distribution of Taiwanese PWID infected with HIV-1 CRF01_AE (*n* = 24), HIV-1 CRF07_BC (*n* = 982), and HCV subtype 6w (*n* = 9) during 2004–2008. The dates of HIV diagnosis and sample collection for most CRF01_AE infections were mainly in 2005 (Figure A2 and Table A2). The CRF07_BC sequences of Taiwanese PWID were grouped into several distinct phylogenetic clusters based on collection places [11] (details shown in Table A2). Based on the dates of HIV diagnosis, our data implied that CRF01_AE started to circulate in the Taiwanese PWID population in Central Taiwan and then spread to other regions of the island. In contrast, CRF07_BC first appeared in the south and moved northward to expand to central-north Taiwan (Figure A2 and Table 1). 

The nine Taiwanese PWID infected with HCV-6w were identified when serving their prison sentences. Among them, five were HCV mono-infections and the other four cases were HIV/HCV coinfections. Six of the cases were from northern Taiwan (Taipei Detention Center, Sindian Drug Abuse Treatment Center and Taoyuan Woman’s Prison), two were from central Taiwan (Taichung Prison and Yunlin Second Prison), and one was from southern Taiwan (Kaohsiung Prison) (Figure A3).

### 3.2. An Estimated Timescale of the Spread of CRF01_AE and CRF07_BC among Taiwanese PWID

When estimating the time scale of the spread of HIV-1 CRF01_AE and CRF07_BC in Asia, we adopted the data set based on GTR + Γ_6_ constant model to pinpoint the time of the most recent common ancestor (tMRCA) of the HIV-1 strains circulating in this area. Compared to the results of the three models, similar conclusions could be reached (Table 1). After systematic analyses, we followed the likelihood of constant size, exponential growth, and the Bayesian skyline model (CRF01_AE: −15,559.7307, −15,548.8808, and −15,719.0827; CRF07_BC: −4939.2117, −4933.7994, and −4957.3370) and found that the exponential growth model was the best to present its transmission.

**Figure 1:**
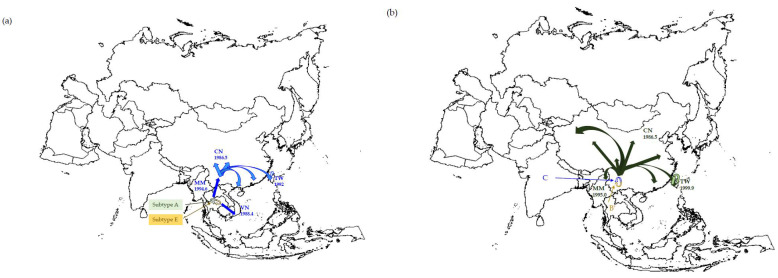

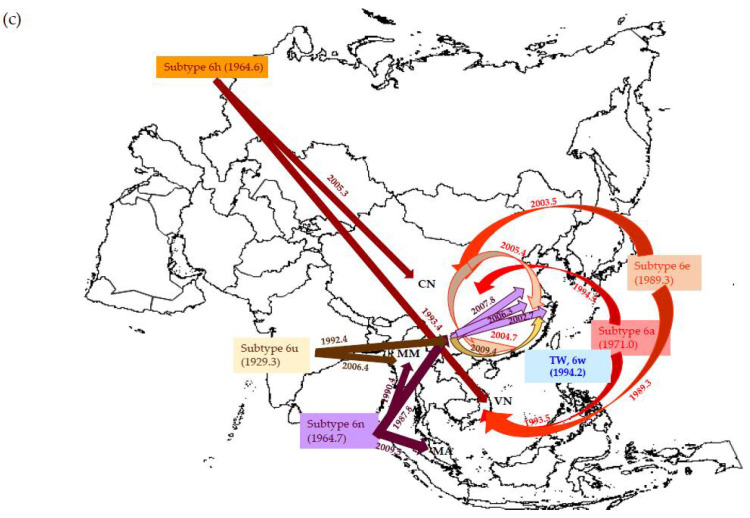
The estimated timescale of the spread of (**a**) HIV-1 CRF01_AE, (**b**) CRF07_BC, and (**c**) HCV genotype-6 in Asian PWID population (CN: China, MM: Myanmar, MA: Malaysia, TW: Taiwan, and VN: Vietnam). In this particular figure, we adopted the data set based on GTR + Γ_6_/GTR + Γ_6_ + I constant model to show the tMRCAs of HIV-1 and HCV strains circulating in this area.

The estimated phylogeny using the *env* gene showed that all Taiwanese CRF01_AE and reference strains formed a single clade. However, there are two different risk groups in Taiwan [11], namely PWID and other sexual groups (e.g., homo, hetero, and bisexuals). The former population that contained sequences from Taipei Detention Center, Taipei Prison, Yunlin Second Prison, and Taichung Prison have a common point. Almost all samples were obtained in 2005 (Table S2). All CRF01_AE strains from Asia (i.e., China, Myanmar, Taiwan, and Vietnam) were dated 1979.0 (95% credible region, CR: 1973.1–1984.0). Concerned about drug addicts, CRF01_AE was introduced to China in 1986.5 (95% CR: 1980.0–1990.8) and then spread to Vietnam in 1988.4 (95% CR: 1984.7–1991.8), Taiwan in 1992, and Myanmar in 1994.6 (95% CR: 1990.2–1999.8). Additionally, CRF01_ AE was first introduced to other Taiwanese sexual groups in 1988.0 (95% CR: 1984.5–1991.1) and then spread to Central Taiwan in 1992.5 (95% CR: 1988.8–1995.9) and Northern Taiwan in 1994.1 (95% CR: 1989.5–1998.4) (Table 1 and Figure 1a). A comparison with our previous findings [11] suggested that CRF01_AE was introduced into Taiwanese PWID through unprotected sex and then caused a local epidemic among PWID through the exchange of injection equipment. 

As summarized in Table 1, all CRF07_BC strains from Asia (i.e., China, Myanmar, and Taiwan) were rooted in 1987.9 (95% CR: 1981.0–1993.9). CRF07_ BC was introduced to China at 1987.9 and spread to Myanmar at 1995.0 (95% CR: 1987.6–2002.0) and to Taiwan at 1999.9 (95% CR: 1997.8–2001.9). Subsequently, this strain spread to other regions of Taiwan in 2001.3 (95% CR: 1998.6–2004.0), 2002.9 (95% CR: 2001.2–2004.2), and 2000.0 (95% CR: 1997.8–2002.2) in Northern, Central, and Southern Taiwan, respectively. The CRF07_BC strains from different regions of Taiwan seem to share a common source that existed in 2000 or thereabouts (95% CR: 1997.8–2001.9) and was part of the Southern Taiwan PWID. This suggests that southern Taiwan was the entry site for CRF07_ BC (Table 1 and Figure 1b). 

### 3.3. An Estimated Timescale of the Spread of HCV Subtype-6w among Taiwanese PWID

Similarly to the estimation of the timescale of HIV-1 spread, we adopted the data set based on GTR + Γ_6_ + I constant model to pinpoint the tMRCAs of HCV-6 and found that the exponential growth model (likelihood in CS: −9744.679, EG: −9717.44, and BS: −9868.01) is the best way to present its transmission. 

Phylogeny analysis using the *NS5B* gene showed that all Asian PWID and reference strains formed a single clade. As summarized in Table 2, all genotype-6 strains from Asia (i.e., China, Myanmar, Taiwan, and Vietnam) were rooted in 1928.1 (95% CR: 1890.2–1966.0). Subtypes 6a, -6n, and -6w had existed in the Taiwanese PWID population (Table A1). Taking subtype-6a for example, it was introduced into Vietnam at 1993.5 (95% CR: 1977.5–2001.3) and later into China at 1994.5 (95% CR: 1988.9–2000.9). Subtype-6n was initially introduced to China (1987.8, 1952.0–2005.0) and then spread to Myanmar (1990.4, 1954.7–2007.7). It is noteworthy that this strain was found to originate in Yunnan (1987.8, 1953.1–2007.0) and spread eastward to Suzhou, Zhenjiang, and Jiangsu in the early and mid-2000s. Furthermore, subtype-6w isolates from different regions in Taiwan seem to share a common source that existed in mid-1990 (95% CR: 1985.0–2001.8) or thereabouts (Table 2 and Figure 1c).

## 4. Discussion

Takebe et al. reported that CRF07_BC strains from different regions in China (including Xinjiang, Liaoning, and probably Guangdong and Sichuan) were likely to share a common ancestor that existed in Yunnan province around 1993 (95% CR: 1991.2–1995.2; *gag*) [13,31]. This suggests that CRF07_BC spreads almost simultaneously to various regions of China [13,31]. Furthermore, CRF07_BC also spread to Taiwan from the South around 1999.7 (95% CR: 1998.2–2001.1; *env*) and spread to the central-north part of Taiwan in 2002.1 (95% CR: 2001.3–2002.9; *env*) [10,13,30], resulting in a major HIV epidemic among PWID in Taiwan [10,11,12,13,31]. The dissemination routes of CRF07_BC in China and Taiwan were those reported in previous studies [11,13,30,31]. To compare the main differences between the use of the date of sample collection versus the date of HIV-1 diagnosis to estimate the time of the emergence of the CRF01_AE and CRF07_BC strains, and to consolidate the integrity of our data, we added more sequences from West Taiwan (e.g., Sindian, Taoyuan, Taichung, Yunlin, and Kaohsiung) in the analysis. As we all know that CRF07_BC circulated in southern Taiwan first, even adding the sequences from the most south area (i.e., Kaohsiung), the tMRCAs of CF07_BC among Taiwanese PWID were behind the estimates as previously reported [13,30,31]. The data obtained using the date of sample collection are more accurate than those using the date of diagnosis. This finding showed why it is necessary to use the correct date to estimate the time of emergence of HIV-1 subtypes or CRFs. Furthermore, our results revealed that the estimated introduction time of CRF01_AE in Taiwan PWID (1992 later) was earlier than that of CRF07_BC (1999.9), and because of the less aggressiveness of CRF01_AE [43], it only caused a local epidemic initially. 

The estimated prevalence of HCV-6 in some regions of Southeast Asia, especially among patients with PWID and major thalassemia, is as high as 50% [44]. Injecting drug abuse is possibly responsible for the high frequency of this genotype in certain parts of Asia. HCV-6 has considerable genetic diversity with 23 subtypes (a–w). HCV-6 infected with HCV-6 respond better to interferon-based therapy compared to genotype 1, although the clinical characteristics and side effect profiles in patients are similar between HCV-6 and other genotypes [44]. Our study showed that HCV-6 was as common as genotype 1 (34.7% vs. 43.5%, Table S1) in the Taiwanese PWID population. According to a large-scale survey on the seroprevalence of HCV in Taiwan [45], the prevalence of injecting drug abuse and incomplete disinfection of medical utensils would cause a small-scale outbreak of HCV in local areas. Furthermore, residents have a higher prevalence of HCV when they were born in an earlier cohort [45]. As shown in Table A1, a cross-sectional study with 624 PWID recruited in Taiwan was conducted in 2007–2008. The overall prevalence of HIV and HCV infection was 44.1% (275/624) and 80.4% (502/624), respectively. The prevalence of HCV mono-infection and HIV/HCV co-infection was 36.4% (227/624) and 44.1% (275/624), respectively. The issues of HCV prevention include the following: to prevent healthy people from being contaminated with infected blood and to avoid reinfection with HCV in cured cases. For those who have been cured and non-infected, regular screening tests are encouraged. Through a series of analyses, our findings appear to support the hypothesis that HCV-6 originated in Southeast Asia (Table 2). HCV-6 is highly divergent from other genotypes and has distinct genetic differences from other strains, suggesting that there may be unclassified subtypes in Asia. Therefore, the accumulation of such genetic heterogeneity suggests that this genotype has circulated, adapted, and evolved in this area for a long period.

There are several limitations to this study. First, all Asian isolates (e.g., China, Malaysia, Myanmar, and Vietnam) were restricted and obtained from the NCBI website. Second, some Asian isolates were excluded from the evolutionary analysis because the sequences were too short or contained missing sequences. Despite the limitations, this study sheds light on the routes of drug trafficking and the resulting high prevalence of HIV-1/HCV coinfections among PWID that could have contributed to regional and global transmissions. In conclusion, for the first time, we report ‘the time of emergence of common HCV and HIV-1 strains among Taiwanese PWID’ and provide a comprehensive profile suggesting the initial circulation of CRF07_BC in southern Taiwan before spreading to other regions of Taiwan. Furthermore, the importance of using the date of sample collection versus the date of HIV-1 diagnosis was also highlighted when estimating the time of the emergence of the CRF01_AE and CRF07_BC strains. Long-term monitoring and implementation in the population at risk would be useful for disease control.

## Figures and Tables

**Table 1:** Evolutionary characteristics of CRF01_AE and CRF07_BC.

HIV-1 CRFs	Genetic Region	GTR + Γ_6_ Constant Size		GTR + Γ_6_ Exponential Growth		GTR + Γ_6_ Bayesian Skyline	
		Rate of Evolution ^a^	tMRCA ^b^	Rate of Evolution	tMRCA	Rate of Evolution	tMRCA
**CRF01_AE**	***env***	**2.6 (1.8–3.5)**		**1.3 (0.9–1.6)**		**7.8 (5.4–10.4)**	
Reference strains			1979.9 (1971.9–1986.1)		1979.5 (1973.9–1984.6)		1988.8 (1987.9–1989.7)
**China (all PWID)**			1989.6 (1985.9–1992.7)		1986.5 (1980.0–1990.8)		1996.1 (1994.2–1997.8)
Guangdong			1996.8 (1992.6–2003.3)		1993.7 (1989.1–1997.9)		2002.9 (2000.8–2005.3)
Guangxi			2000.3 (1993.8–2004.2)		1996.1 (1991.3–2000.0)		2003.8 (2001.0–2005.4)
Guizhou			2000.8 (1994.4–2004.4)		1996.2 (1991.7–2000.3)		2004.1 (2001.0–2005.5)
Sichuan			2004.5 (2003.2–2005.6)		2001.0 (1998.3–2003.6)		2005.5 (2005.0–2005.9)
Yunnan			1989.7 (1986.0–1993.0)		1986.5 (1980.0–1990.9)		1996.7 (1994.6–2001.2)
Myanmar (all PWID)			1999.2 (1995.5–2002.5)		1994.6 (1990.2–1999.8)		2002.2 (2000.4–2004.5)
**Taiwan (all)**			1989.9 (1987.0–1992.3)		1988.0 (1984.5–1991.1)		1996.0 (1991.8–1997.8)
PWID, Central Taiwan			1996.3 (1993.0–1999.7)		1992.5 (1988.8–1995.9)		2001.2 (2000.3–2001.6)
PWID, Northern Taiwan			1999.0 (1995.5–2002.4)		1994.1 (1989.5–1998.4)		2001.5 (2000.6–2003.5)
Other sexual-groups			1989.9 (1987.0–1992.3)		1988.0 (1984.5–1991.1)		1996.0 (1991.8–1997.8)
Vietnam (all PWID)			1990.5 (1987.4–1993.3)		1988.4 (1984.7–1991.8)		1994.1 (1991.9–1996.0)
**CRF07_BC**	***env***	**1.4 (0.6–2.3)**		**1.3 (0.7–1.9)**		**5.3 (2.8–8.3)**	
Reference strains			1992.0 (1986.5–1995.7)		1994.1 (1990.8–1996.0)		1995.7 (1994.6–1996.0)
China (all PWID)			1970.6 (1943.5–1989.8)		1987.9 (1981.0–1993.9)		1994.3 (1992.2–1995.6)
Gansu			1998.9 (1994.5–2001.9)		1999.7 (1996.4–2001.9)		2000.3 (1997.2–2001.9)
Guangdong			1976.1 (1954.6–1992.2)		1989.3 (1981.5–1997.1)		1997.1 (1996.4–1997.8)
Ningxia			1976.4 (1955.9–1991.8)		1989.3 (1981.9–1997.0)		1997.0 (1996.2–1997.7)
Qinghai			1993.9 (1986.6–2002.1)		1995.7 (1991.9–2000.5)		1999.0 (1996.6–2004.4)
Sichuan			1986.2 (1974.3–1994.2)		1991.5 (1985.5–1996.4)		1997.0 (1996.2–1997.7)
Xinjiang			1997.9 (1992.0–2004.9)		1999.0 (1994.4–2003.8)		2001.3 (1996.9–2005.6)
Yunnan			1970.7 (1943.6–1989.9)		1987.9 (1981.0–1993.9)		1994.3 (1992.2–1995.6)
Myanmar (all PWID)			1980.9 (1949.6–2004.3)		1995.0 (1987.6–2002.0)		1997.1 (1996.2–1997.9)
Taiwan (all PWID)			1999.7 (1997.0–2002.4)		1999.9 (1997.8–2001.9)		2001.7 (1999.8–2003.5)
Central Taiwan			2003.3 (2001.9–2004.6)		2002.9 (2001.2–2004.2)		2004.2 (2003.5–2005.0)
Northern Taiwan			2001.9 (1998.3–2004.0)		2001.3 (1998.6–2004.0)		2003.7 (2002.6–2004.9)
Southern Taiwan			1999.8 (1997.0–2002.3)		2000.0 (1997.8–2002.2)		2001.7 (1999.8–2003.5)

^a^ Rates are expressed as 10^−3^ nucleotide substitutions per site per year. 95% highest posterior density (HPD) confidence intervals are shown in parenthesis. ^b^ tMRCA, Time of the most recent common ancestor. 95% HPDs are shown in parenthesis.

**Table 2:** Evolutionary characteristics of HCV subtype 6w.

	Genetic Region	GTR + Γ_6_ + I Constant Size		GTR + Γ_6_ + I Exponential Growth		GTR + Γ_6_ + I Bayesian Skyline	
		Rate of Evolution ^a^	tMRCA ^b^	Rate of Evolution	tMRCA	Rate of Evolution	tMRCA
**Genotype-6 (all)**	***NS5B***	1.3 (1.0–1.7)	1916.6 (1866.9–1956.9)	1.0 (0.6–1.4)	1928.1 (1890.2–1966.0)	3.6 (2.0–6.0)	1973.2 (1951.0–1989.3)
Reference strains			1920.4 (1870.5–1959.3)		1928.4 (1890.3–1966.0)		1974.1 (1949.0–1989.9)
**Subtype 6a (all)**			1966.0 (1929.6–1987.2)		1971.0 (1947.4–1988.4)		1990.4 (1984.5–1994.8)
Reference strains			1966.1 (1926.3–1987.1)		1971.0 (1947.4–1988.4)		1990.4 (1984.5–1994.8)
China (PWID)			1995.8 (1991.3–2000.5)		1994.5 (1988.9–2000.9)		2000.0 (1995.4–2003.6)
Vietnam (PWID)			1994.9 (1989.7–2001.7)		1993.5 (1977.5–2001.3)		1999.3 (1995.2–2004.9)
**Subtype 6e (all)**			1991.3 (1983.3–1998.6)		1989.3 (1978.8–1997.6)		1998.0 (1993.6–2001.4)
Reference strains			1991.3 (1983.3–1998.6)		1989.3 (1978.8–1997.6)		1998.2 (1994.0–2003.0)
China (PWID)			2003.7 (2000.5–2005.8)		2003.5 (1999.4–2005.7)		2003.3 (2002.4–2005.9)
Hong Kong			2004.8 (2002.3–2005.9)		2004.7 (2002.0–2005.9)		2004.9 (2003.0–2006.0)
Zhenjiang			2005.4 (2003.6–2007.2)		2005.1 (2002.6–2006.8)		2006.2 (2002.9–2007.6)
Vietnam (PWID)			1991.3 (1982.9–1998.3)		1989.3 (1979.5–1998.4)		1999.3 (1993.6–2003.0)
**Subtype 6h (all)**			1965.3 (1940.6–1984.7)		1964.6 (1928.2–1987.9)		1982.6 (1962.3–1993.6)
Reference strains			1993.9 (1990.0–1996.0)		1993.8 (1990.2–1996.0)		1994.8 (1992.5–1996.0)
China (PWID)			2006.0 (n/a)		2005.3 (n/a)		2006.0 (n/a)
Vietnam (PWID)			1994.0 (1981.7–1996.0)		1993.4 (1987.2–1996.0)		1998.4 (1993.5–2006.0)
**Subtype 6n (all)**			1965.9 (1941.6–1984.7)		1964.7 (1929.5–1988.3)		1983.7 (1963.1–1993.6)
Reference strains			1979.1 (1946.8–2001.1)		1975.7 (1934.5–2001.3)		1983.7 (1963.1–1993.6)
China (PWID)			1984.7 (1950.8–2004.8)		1987.8 (1952.0–2005.0)		2003.2 (2000.6–2005.0)
Jiangsu			2008.2 (2006.7–2009.8)		2007.8 (2006.1–2009.6)		2009.2 (2008.2–2010.0)
Suzhou			2003.7 (2000.9–2007.4)		2002.7 (1998.8–2007.3)		2004.7 (2002.8–2008.2)
Yunnan			1984.7 (1950.8–2004.8)		1987.8 (1953.1–2007.0)		2003.2 (2000.4–2005.0)
Zhenjiang			2006.8 (2004.3–2008.5)		2006.3 (2003.3–2008.3)		2007.9 (2006.8–2008.8)
Myanmar (PWID)			1985.1 (1951.2–2006.5)		1990.4 (1954.7–2007.7)		2004.1 (2000.7–2008.1)
Malaysia (PWID)			2010.9 (2009.0–2012.9)		2009.5 (2005.7–2013.1)		2011.8 (2009.3–2013.7)
**Subtype 6r**							
Reference strains			1974.6 (1945.1–1994.2)		1976.1 (1955.8–1993.0)		1997.9 (1993.8–2001.6)
**Subtype 6v**							
Reference strains			1954.6 (1910.7–1984.7)		1961.5 (1925.2–1987.7)		1988.2 (1972.4–1998.9)
**Subtype 6u (all)**			1918.3 (1867.2–1957.7)		1929.3 (1892.6–1967.3)		1976.3 (1957.3–1990.2)
Reference strains			1918.3 (1967.2–1957.7)		1929.3 (1892.6–1967.3)		1976.4 (1957.9–1993.7)
China (PWID)			1999.4 (1997.5–2000.9)		1992.4 (1958.7–2001.0)		1999.2 (1990.5–2001.0)
Suzhou			2009.7 (2008.3–2010.8)		2009.4 (2007.9–2010.7)		2010.4 (2009.3–2011.0)
Yunnan			1999.5 (1997.5–2000.9)		1992.4 (1958.7–2001.0)		1999.2 (1990.5–2001.0)
Myanmar (PWID)			2007.2 (1999.4–2010.3)		2006.4 (1998.9–2009.9)		2009.2 (2007.7–2011.2)
**Subtype 6w**							
Reference strains			2000.4 (1995.2–2004.0)		1999.8 (1993.6–2009.8)		2002.5 (2000.2–2004.5)
Taiwan (PWID) ^c^			1996.7 (1990.0–2002.2)		1994.2 (1985.0–2001.8)		2000.0 (1987.6–2004.2)

^a^ Rates are expressed as 10^−3^ nucleotide substitutions per site per year. 95% highest posterior density (HPD) confidence intervals are shown in parenthesis. ^b^ tMRCA, Time of the most recent common ancestor. 95% HPDs are shown in parenthesis. If there were extreme values for estimating shown “unavailable, n/a”. ^c^ Nine participants recruited in different parts of Taiwan. Sample collection from Taipei Detention Center, Sindian Drug Abuser Treatment Center, Taoyuan Women’s Prison, Taichung Prison, and Kaohsiung Prison were 1, 2, 3, 2, and 1, respectively.

## Data Availability

The data presented in this study are available in [Section 2.4 and Table A2]. Part of this paper was presented at the 8th Second Member Conference and Academic Symposium held in Taiwan in 2020.

## Appendix A

**Table A1:** Distribution of HCV genotypes/subtypes in the different groups among Taiwanese PWID.

	2004–2006 HIV-1/HCV Co-Infection (*n* = 165) ^a^	2007–2008 HIV-1/HCV Co-Infection (*n* = 275) ^b^	2007–2008 HCV Mono-Infection (*n* = 227) ^b^	*p*-Value
Single infection				<0.001
1a	21 (12.7)	64 (23.3)	53 (23.3)	
1b	48 (29.1)	25 (9.1)	30 (13.2)	
2a	6 (3.6)	10 (3.6)	6 (2.6)	
2b	6 (3.6)	21 (7.6)	5 (2.2)	
3a	11 (6.7)	24 (8.7)	25 (11.0)	
3b	1 (0.6)	3 (1.1)	3 (1.3)	
6a	43 (26.1)	80 (29.1)	53 (23.3)	
6n	4 (2.4)	1 (0.4)	3 (1.3)	
6w	1 (0.6)	2 (0.7)	5 (2.2)	
Subtotal_1	141 (85.5)	230 (83.6)	183 (80.6)	
Double infection				<0.001
1a/1b	5 (3.0)	5 (1.8)	0 (0.0)	
1a/2a	0 (0.0)	4 (1.5)	1 (0.4)	
1a/2b	0 (0.0)	18 (6.5)	5 (2.2)	
1a/3a	0 (0.0)	1 (0.4)	2 (0.9)	
1a/6a	1 (0.6)	0 (0.0)	1 (0.4)	
1b/2a	1 (0.6)	0 (0.0)	0 (0.0)	
1b/2b	1 (0.6)	0 (0.0)	0 (0.0)	
1b/3a	3 (1.8)	0 (0.0)	0 (0.0)	
1b/3b	0 (0.0)	1 (0.4)	0 (0.0)	
1b/6a	6 (3.6)	4 (1.5)	6 (2.6)	
1b/6w	1 (0.6)	0 (0.0)	0 (0.0)	
2a/2b	0 (0.0)	1 (0.4)	1 (0.4)	
2a/3a	0 (0.0)	0 (0.0)	3 (1.3)	
2a/6a	0 (0.0)	8 (2.9)	20 (8.8)	
2b/6a	0 (0.0)	2 (0.7)	1 (0.4)	
3a/6a	3 (1.8)	1 (0.4)	2 (0.9)	
6a/6n	1 (0.6)	0 (0.0)	0 (0.0)	
Subtotal_2	22 (13.3)	45 (16.4)	42 (18.5)	
Triple infection				0.261
1a/1b/3a	1 (0.6)	0 (0.0)	0 (0.0)	
1a/1b/6a	1 (0.6)	0 (0.0)	0 (0.0)	
1a/2a/3a	0 (0.0)	0 (0.0)	1 (0.4)	
1b/3a/6a	0 (0.0)	0 (0.0)	1 (0.4)	
Subtotal_3	2 (1.2)	0 (0.0)	2 (0.9)	

^a^ 180 blood samples randomly selected by the following strains (50 HIV-1 CRF07_BC-infected cases each from northern, central, and southern regions of Taiwan; 20 HIV-1 subtype B-infected cases; 10 HIV-1 CRF01_AE-infected cases) in years 2004–2006, among which 91.7% (165/180) were completed genotype identified by RT-PCR and phylogenetic analysis. ^b^ A total of 624 blood specimens were collected in the years 2007–2008; among them, 80.4% (502/624) were completed genotype identified by RT-PCR and phylogenetic analysis.

**Table A2:** Location and collection date of Asian isolates from PWID infected with HIV-1 CRF01_AE, CRF07_BC, and HCV-6 and other related reference strains from GenBank.

Infections	Accession Numbers/Sample ID	Location	Collection Date
HIV-1 CRF01_AE (reference strains)	90CF11697/F197340	Central African Republic	1990
	90CF4071/AF197341	Central African Republic	1990
	90CR402/U51188	Central African Republic	1990
HIV-1 CRF01_AE ^a^ (Asian PWID)	HM215409	Yunnan	2007
	HM215410	Yunnan	2006
	HM215418	Yunnan	2007
	HM215419	Yunnan	2007
	HM215422	Yunnan	2006
	HM215427	Yunnan	2006
	JN223054	Myanmar	2009
	JX112823	Guangdong	8 Nov 2007
	JX112824	Guangdong	23 Jul 2007
	JX112825	Guangdong	23 Jul 2007
	JX112826	Guangdong	4 Jul 2007
	JX112828	Guangdong	11 Jul 2007
	JX112831	Guangxi	22 May 2007
	JX112837	Guangxi	22 Oct 2007
	JX112839	Guangxi	8 Nov 2007
	JX112840	Guizhou	19 Jul 2007
	JX112842	Guizhou	6 Sep 2007
	JX112857	Sichuan	3 Dec 2006
	JX112861	Yunnan	4 Jun 2002
	JX112863	Yunnan	4 Jun 2002
	KP401977	Viet Nam	6 Jan 2012
	KP401980	Viet Nam	9 Jan 2012
	KP401983	Viet Nam	9 Jan 2012
	KP401984	Viet Nam	9 Jan 2012
	KP401987	Viet Nam	10 Jan 2012
	KP401992	Viet Nam	11 Jan 2012
	KP401995	Viet Nam	12 Jan 2012
	KP401997	Viet Nam	11 Jan 2012
	KP401998	Viet Nam	7 Feb 2012
	KP402000	Viet Nam	8 Feb 2012
	KP402001	Viet Nam	8 Feb 2012
	KP402003	Viet Nam	8 Feb 2012
	KP402004	Viet Nam	8 Feb 2012
	KP402005	Viet Nam	9 Feb 2012
	KP402009	Viet Nam	13 Feb 2012
	KP402010	Viet Nam	13 Feb 2012
	KP402014	Viet Nam	15 Feb 2012
	KP402015	Viet Nam	16 Feb 2012
	KP402017	Viet Nam	16 Feb 2012
	KP402019	Viet Nam	20 Feb 2012
	KP402020	Viet Nam	20 Feb 2012
	KP402021	Viet Nam	20 Feb 2012
	KP402022	Viet Nam	21 Feb 2012
	KP402024	Viet Nam	21 Feb 2012
	KP402026	Viet Nam	21 Feb 2012
	KP402027	Viet Nam	22 Feb 2012
	KP402030	Viet Nam	22 Feb 2012
HIV-1 CRF01_AE ^a^ (Asian PWID)	KP402031	Viet Nam	22 Feb 2012
	KP402037	Viet Nam	28 Feb 2012
	KP402039	Viet Nam	9 Mar 2012
	KP402040	Viet Nam	9 Mar 2012
	KP402043	Viet Nam	14 Mar 2012
	KP402044	Viet Nam	15 Mar 2012
	KP402045	Viet Nam	28 Mar 2012
	KP402046	Viet Nam	29 Mar 2012
	KP402050	Viet Nam	16 Apr 2012
	KP402051	Viet Nam	10 May 2012
	KP402052	Viet Nam	15 May 2012
	KP402056	Viet Nam	29 Feb 2012
	KU820849	Myanmar	8 Nov 2014
Group 1 (Taiwanese PWID only)	D212	Taipei Detention Center	4 May 2005
	D261	Taipei Prison	18 May 2005
	D291	Yunlin Second Prison	9 Jun 2005
	D299	Yunlin Second Prison	9 Jun 2005
	D301	Yunlin Second Prison	9 Jun 2005
	D308	Yunlin Second Prison	9 Jun 2005
	D336	Yunlin Second Prison	31 Aug 2005
	D370	Yunlin Second Prison	31 Aug 2005
	D376	Yunlin Second Prison	31 Aug 2005
	D412	Taipei Detention Center	6 Oct 2005
	D423	Taipei Detention Center	6 Oct 2005
	D455	Taipei Detention Center	6 Oct 2005
	D568	Yunlin Second Prison	22 Dec 2005
	D580	Yunlin Second Prison	22 Dec 2005
	D737	Taichung Prison	26 Mar 2008
Group 2 (Taiwanese other sexual-groups, e.g., homo-, hetero-, and bisexuals)	4V370	Taipei City Hospital	7 Jan 2005
	4V396	Taipei City Hospital	14 Feb 2005
	4V428	Taipei City Hospital	17 Mar 2005
	4V481	Taipei City Hospital	30 May 2005
	4V503	Taipei City Hospital	14 Jul 2005
	4V507	Taipei City Hospital	20 Jul 2005
	4V545	Taipei City Hospital	13 Sep 2005
	4V569	Taipei City Hospital	4 Oct 2005
	4V590	Taipei City Hospital	28 Oct 2005
	4V614	Taipei City Hospital	11 Nov 2005
	4V680	Taipei City Hospital	3 Jan 2006
	4V689	Taipei City Hospital	4 Jan 2006
	4V691	Taipei City Hospital	12 Jan2006
	4V714	Taipei City Hospital	24 Feb 2006
	4V724	Taipei City Hospital	20 Feb 2006
	4V740	Taipei City Hospital	7 Mar 2006
	4V744	Taipei City Hospital	9 Mar 2006
	4V795	Taipei City Hospital	9 May 2006
	4V872	Taipei City Hospital	21 Aug 2006
HIV-1 CRF07_BC (reference strains)	AF286226/97CN001	China	1997
	/C54A	China	1997
	/C54D	China	1997
	/C54	China	1997
	/CN54	China	1997
	AF286230/98CN009	China	1998
	AF503396/CNGL179	China	-
HIV-1 CRF07_BC ^a^ (Asian PWID)	JN223069	Myanmar	2009
	JX392363	Gansu	2002
	JX392364	Ningxia	2002
	JX392365	Ningxia	2002
	JX392366	Ningxia	2002
	JX392367	Ningxia	2005
	JX392368	Qinghai	2006
	JX392369	Qinghai	2005
	JX392370	Sichuan	1999
	JX392371	Sichuan	1998
	JX392372	Sichuan	1999
	JX392373	Sichuan	2003
	JX392374	Sichuan	2002
	JX392375	Yunnan	1996
	JX392376	Yunnan	1997
	JX392377	Yunnan	2001
	JX392378	Sichuan	2006
	JX392379	Sichuan	2006
	JX392380	Sichuan	2006
	JX392381	Sichuan	2006
	JX392382	Sichuan	2006
	JX392384	Xinjiang	2006
	KF250368	Guangdong	2007
	KF250369	Guangdong	2007
	KF250370	Guangdong	2007
	KF250371	Guangdong	2007
	KF250375	Ningxia	2007
	KF250376	Sichuan	2006
	KF250377	Xinjiang	2007
	KF250378	Yunnan	1996
	KF250379	Yunnan	1996
	KF250380	Yunnan	1996
	KU820832	Myanmar	28 Nov 2013
Group 1 (Taiwanese PWID only)	D74	Tainan Detention Center	30 Dec 2004
	D75	Tainan Detention Center	30 Dec 2004
	D76	Tainan Detention Center	30 Dec 2004
	D97	Tainan Detention Center	30 Dec 2004
	D114	Tainan Detention Center	30 Dec 2004
	D118	Tainan Detention Center	30 Dec 2004
	D120	Tainan Detention Center	30 Dec 2004
Group 2 (Taiwanese PWID only)	4V780	Taipei City Hospital	27 Apr 2006
	4V784	Taipei City Hospital	24 Apr 2006
	4V793	Taipei City Hospital	8 May 2006
	4V807	Taipei City Hospital	17 May 2006
	4V844	Taipei City Hospital	11 Jul 2006
	D126	Tainan Detention Center	30 Dec 2004
	D338	Yunlin Second Prison	31 Aug 2005
	D734	Taichung Prison	26 Mar 2008
	D757	Taichung Prison	26 Mar 2008
	D776	Taichung Prison	26 Mar 2008
Group 3 (Taiwanese PWID only)	4V457	Taipei City Hospital	27 Apr 2005
	4V467	Taipei City Hospital	9 May 2005
	D733	Taichung Prison	26 Mar 2008
	D735	Taichung Prison	26 Mar 2008
	D736	Taichung Prison	26 Mar 2008
	D771	Taichung Prison	26 Mar 2008
	D775	Taichung Prison	26 Mar 2008
	D780	Taichung Prison	26 Mar 2008
	D839	Taichung Prison	26 Mar 2008
	D845	Taichung Prison	26 Mar 2008
	D850	Kaohsiung Prison	9 Apr 2008
	D863	Kaohsiung Prison	9 Apr 2008
	D902	Kaohsiung Prison	9 Apr 2008
	D907	Kaohsiung Prison	9 Apr 2008
	D947	Kaohsiung Prison	9 Apr 2008
	D966	Taoyuan Woman’s Prison	6 May 2008
	D970	Taoyuan Woman’s Prison	6 May 2008
	D1007	Taoyuan Woman’s Prison	6 May 2008
HCV-6 (reference strains)	DQ278892/GZ52557	China	2002
	KC191671/10MYKJ032	Malaysia	2010
Subtype 6a (reference strains)	HQ912954/PR58	China	2008
	HQ912955/PR144	China	2008
	KC844037/ZS221	China	2009
	KC844038/ZS674	China	2011
	AY859526/6a33	Hong Kong	2004
	AY973865/cs6a-16	Hong Kong	2004
	AY973866/cs6a-18	Hong Kong	2004
	DQ480512/6a77	Hong Kong	2004
	DQ480513/6a35	Hong Kong	2004
	DQ480514/6a63	Hong Kong	2004
	DQ480515/6a64	Hong Kong	2004
	DQ480516/6a61	Hong Kong	2004
	DQ480517/6a73	Hong Kong	2004
	DQ480518/6a65	Hong Kong	2004
	DQ480519/6a66	Hong Kong	2004
	DQ480520/6a67	Hong Kong	2004
	DQ480521/6a69	Hong Kong	2004
	DQ480522/6a72	Hong Kong	2004
	DQ480523/6a62	Hong Kong	2004
	DQ480524/6a74	Hong Kong	2004
	Y12083/EUHK2	Hong Kong	1997
	EU246930/D9	Viet Nam	-
Subtype 6a (Asian PWID)	JX102891	Viet Nam	22 Aug 2008
	JX102895	Viet Nam	30 Aug 2008
	JX102896	Viet Nam	30 Aug 2008
	JX102897	Viet Nam	30 Aug 2008
	JX102908	Viet Nam	31 Aug 2008
	JX102957	Viet Nam	21 May 2008
	JX102963	Viet Nam	21 Feb 2008
	JX102965	Viet Nam	21 May 2008
	JX102973	Viet Nam	21 May 2008
	JX102975	Viet Nam	29 May 2008
	JX103004	Viet Nam	5 Sep 2008
	JX103010	Viet Nam	15 Oct 2008
	JX103038	Viet Nam	28 Jun 2009
	JX103047	Viet Nam	1 Jul 2009
	JX103051	Viet Nam	3 Jul 2009
	JX103104	Viet Nam	7 Sep 2009
	JX103106	Viet Nam	7 Sep 2009
	JX103117	Viet Nam	9 Sep 2009
	JF721080	Guangdong	15 Nov 2010
	JF721081	Guangdong	15 Nov 2010
	JF721082	Guangdong	15 Nov 2010
	JF721083	Guangdong	15 Nov 2010
	JF721084	Guangdong	15 Nov 2010
	JF721085	Guangdong	15 Nov 2010
	JF721086	Guangdong	15 Nov 2010
Subtype 6a (Asian PWID)	JF721087	Guangdong	15 Nov 2010
	JF721088	Guangdong	15 Nov 2010
	JF721089	Guangdong	15 Nov 2010
	JF721090	Guangdong	15 Nov 2010
	JF721091	Guangdong	15 Nov 2010
	JF721092	Guangdong	15 Nov 2010
	JF721093	Guangdong	15 Nov 2010
	JF721094	Guangdong	15 Nov 2010
	JF721095	Guangdong	15 Nov 2010
	JF721096	Guangdong	15 Nov 2010
	JF721097	Guangdong	15 Nov 2010
	JF721098	Guangdong	15 Nov 2010
	JF721099	Guangdong	15 Nov 2010
	JF721100	Guangdong	15 Nov 2010
	JF721101	Guangdong	15 Nov 2010
	JF721102	Guangdong	15 Nov 2010
	JF721103	Guangdong	15 Nov 2010
	JF721104	Guangdong	15 Nov 2010
	JF721105	Guangdong	15 Nov 2010
	JF721106	Guangdong	15 Nov 2010
	JF721107	Guangdong	15 Nov 2010
	JF721108	Guangdong	15 Nov 2010
	JF721109	Guangdong	15 Nov 2010
	JF721110	Guangdong	15 Nov 2010
	JF721111	Guangdong	15 Nov 2010
	JF721112	Guangdong	15 Nov 2010
	JF721113	Guangdong	15 Nov 2010
	JF721114	Guangdong	15 Nov 2010
	JF721115	Guangdong	15 Nov 2010
	JF721116	Guangdong	15 Nov 2010
	JF721117	Guangdong	15 Nov 2010
	JF721118	Guangdong	15 Nov 2010
	JF721119	Guangdong	15 Nov 2010
	JF721120	Guangdong	15 Nov 2010
	JF721121	Guangdong	15 Nov 2010
	JF721122	Guangdong	15 Nov 2010
	JF721123	Guangdong	15 Nov 2010
	JF721124	Guangdong	15 Nov 2010
	JF721125	Guangdong	15 Nov 2010
	JF721126	Guangdong	15 Nov 2010
	JF721127	Guangdong	15 Nov 2010
	JF721128	Guangdong	15 Nov 2010
	JF721129	Guangdong	15 Nov 2010
	JF721130	Guangdong	15 Nov 2010
	JF721131	Guangdong	15 Nov 2010
	JF721132	Guangdong	15 Nov 2010
	JF721133	Guangdong	15 Nov 2010
	JF721134	Guangdong	15 Nov 2010
	JF721135	Guangdong	15 Nov 2010
	JF721136	Guangdong	15 Nov 2010
Subtype 6a (Asian PWID)	JF721137	Guangdong	15 Nov 2010
	JF721138	Guangdong	15 Nov 2010
	JF721139	Guangdong	15 Nov 2010
	JF721140	Guangdong	15 Nov 2010
	JF721141	Guangdong	15 Nov 2010
	JF721142	Guangdong	15 Nov 2010
	JF721143	Guangdong	15 Nov 2010
	JF721144	Guangdong	15 Nov 2010
	JF721145	Guangdong	15 Nov 2010
	JF721146	Guangdong	15 Nov 2010
	JF721147	Guangdong	15 Nov 2010
	JF721148	Guangdong	15 Nov 2010
	JF721149	Guangdong	15 Nov 2010
	JF721150	Guangdong	15 Nov 2010
	JF721151	Guangdong	15 Nov 2010
	JF721152	Guangdong	15 Nov 2010
	JF721153	Guangdong	15 Nov 2010
	JF721154	Guangdong	15 Nov 2010
	JF721155	Guangdong	15 Nov 2010
	JF721156	Guangdong	15 Nov 2010
	JF721157	Guangdong	15 Nov 2010
Subtype 6e (reference strains)	DQ314805/GX004	China	-
	LC435023/N12-2804-Cam	Cambodia	22 Aug 2012
	LC435027/N16-2804-Cam	Cambodia	3 Sep 2016
	EU246931/D42	Viet Nam	-
	EU246932/D88	Viet Nam	-
	EU408326/537798	USA	-
Subtype 6e (Asian PWID)	AB523168	Viet Nam	2007
	AB523178	Viet Nam	2007
	AB523179	Viet Nam	2007
	AB523190	Viet Nam	2007
	AB523248	Viet Nam	2007
	AB523321	Viet Nam	2007
	AB523326	Viet Nam	2007
	AB523330	Viet Nam	2007
	AB523339	Viet Nam	2007
	AB523345	Viet Nam	2007
	AB523346	Viet Nam	2007
	HM009307	Hong Kong	Jan 2006
	HQ318922	Zhenjiang	2009
	HQ318923	Zhenjiang	2009
	HQ318924	Zhenjiang	2009
	HQ318925	Zhenjiang	2009
	HQ318926	Zhenjiang	2009
	HQ318927	Zhenjiang	2009
	JX102902	Viet Nam	30 Aug 2008
	JX102969	Viet Nam	21 May 2008
	JX102982	Viet Nam	29 May 2008
	JX103102	Viet Nam	10 Jul 2009
	JX103119	Viet Nam	10 Sep 2009
Subtype 6h (reference strains)	D84265/VN004	Viet Nam	-
Subtype 6h (Asian PWID)	AB523188	Viet Nam	2007
	AB523257	Viet Nam	2007
	AB523259	Viet Nam	2007
	AB523289	Viet Nam	2007
	AB523290	Viet Nam	2007
	AB523312	Viet Nam	2007
	AB523332	Viet Nam	2007
	HM009308	Hong Kong	2006
	JX102967	Viet Nam	21 May 2008
	JX103044	Viet Nam	1 Jul 2009
Subtype 6n (reference strains)	DQ278894/KM42	China	2002
	DQ835768/D86/93	Thailand	-
	EU246937/TH22	Thailand	-
	EU246938/TH31	Thailand	-
Subtype 6n (Asian PWID)	HQ318920	Zhenjiang	2009
	HQ318921	Zhenjiang	2009
	JQ303547	Suzhou	2011
	JQ303548	Suzhou	2011
	JQ303549	Suzhou	2011
	JQ303550	Suzhou	2011
	JQ303551	Suzhou	2010
	JQ303552	Suzhou	2010
	JQ303553	Suzhou	2011
	JQ303554	Suzhou	2010
	KC878938	Jiangsu	2011
	KC878983	Jiangsu	2011
	KM285079	Yunnan	2011
	KM285080	Yunnan	2011
	KM285081	Yunnan	2012
	KM285082	Yunnan	2012
	KM285083	Yunnan	2012
	KM285084	Yunnan	2009
	KM285085	Yunnan	2010
	KM285086	Yunnan	2012
	KM285087	Yunnan	2009
	KM285088	Yunnan	2012
	KM285089	Yunnan	2012
	KM285090	Yunnan	2012
	KM285091	Yunnan	2012
	KM285092	Yunnan	2012
	KM285093	Yunnan	2012
	KM285094	Yunnan	2010
	KM285095	Yunnan	2010
	KM285096	Yunnan	2011
	KM285097	Yunnan	2009
	KM285098	Yunnan	2011
	KR108496	Malaysia	2009
	KR108497	Malaysia	2009
Subtype 6n (Asian PWID)	KT735662	Yunnan	2014
	KT735668	Yunnan	2014
	KT735681	Yunnan	2014
	KT735687	Yunnan	2014
	KT735689	Yunnan	2014
	KT735695	Yunnan	2014
	KT735697	Yunnan	2014
	KT735698	Yunnan	2014
	KT735704	Yunnan	2014
	KT735709	Yunnan	2014
	KT735715	Yunnan	2014
	KT735718	Yunnan	2014
	KT735723	Yunnan	2014
	KT735726	Yunnan	2014
	KT735727	Yunnan	2014
	KT735732	Yunnan	2014
	KT735733	Yunnan	2014
	KT735736	Yunnan	2014
	KT735743	Yunnan	2014
	KT735759	Yunnan	2014
	KT735779	Yunnan	2014
	KT735804	Yunnan	2014
	KT735814	Yunnan	2014
	KT735820	Yunnan	2014
	KT735821	Yunnan	2014
	KT735822	Yunnan	2014
	KT735827	Yunnan	2014
	KT735834	Yunnan	2014
	KT735855	Yunnan	2014
	KT735878	Yunnan	2014
	MH458979	Myanmar	2014
	MH458983	Myanmar	2014
	MH458986	Myanmar	2014
	MH458995	Myanmar	2014
Subtype 6r (reference strains)	EU408328/QC245	Canada	-
	LC435024/N12-2911-Cam	Cambodia	22 Aug 2012
	LC435028/N16-2911-Cam	Cambodia	3 Sep 2016
Subtype 6v (reference strains)	EU158186/NK46	China	2004
	EU798760/KMN-02	China	-
	EU798761/KM046	China	-
	FJ435090/KM181	China	-
Subtype 6u (reference strains)	EU246940/D83	Viet Nam	-
	EU408330/DH012	China	2001
	EU408331/DH014	China	2001
	EU408332/DH028	China	2001
Subtype 6u (Asian PWID)	JQ303555	Suzhou	2011
	KM285099	Yunnan	2012
	KM285100	Yunnan	2009
	KM285101	Yunnan	2012
	KM285102	Yunnan	2009
Subtype 6u (Asian PWID)	KM285103	Yunnan	2011
	KM285104	Yunnan	2009
	KM285105	Yunnan	2011
	KM285106	Yunnan	2011
	KM285107	Yunnan	2011
	KM285108	Yunnan	2011
	KM285109	Yunnan	2009
	KM285110	Yunnan	2012
	KM285111	Yunnan	2011
	KM285112	Yunnan	2011
	KM285113	Yunnan	2010
	KM285114	Yunnan	2009
	KM285115	Yunnan	2012
	KM285116	Yunnan	2012
	KM285117	Yunnan	2011
	KM285118	Yunnan	2009
	KT735674	Yunnan	2014
	KT735690	Yunnan	2014
	KT735700	Yunnan	2014
	KT735712	Yunnan	2014
	KT735744	Yunnan	2014
	KT735810	Yunnan	2014
	MH458978	Myanmar	2014
	MH458980	Myanmar	2014
	MH458984	Myanmar	2014
	MH458993	Myanmar	2014
Subtype 6w (reference strains)	EU643834/HCV-6-D140	Taiwan	2005
	EU643836/HCV-6-D370	Taiwan	2005
HCV subtype 6w (Taiwanese PWID only)	D140	Taipei Detention Center	20 Jan 2005
	D370	Yunlin Second Prison	31 Aug 2005
	D778	Taichung Prison	26 Mar 2008
	D866	Kaohsiung Prison	9 Apr 2008
	ND141	Taoyuan Woman’s Prison	27 May 2008
	ND153	Taoyuan Woman’s Prison	27 May 2008
	ND187	Taoyuan Woman’s Prison	27 May 2008
	ND414	Sindian Drug Abuser Treatment Center	18 Jun 2008
	ND415	Sindian Drug Abuser Treatment Center	18 Jun 2008

Note: ^a^ The lengths of the *env* fragment in CRF01_AE and CRF07_BC were up to 537 and 552 bp, respectively. The length of the query shorter than the standard was labeled with a different color, such as ‘light gray, <500 bp’ and ‘dark gray, <300 bp’.
